# Veterinary World reviewer acknowledgment 2025

**DOI:** 10.14202/vetworld.2026.165-168

**Published:** 2026-01-14

**Authors:** A. V. Sherasiya, Riyaz

**Affiliations:** Veterinary World, 208/231, Madras Street, Christchurch Central, Canterbury, New Zealand

## Contributing reviewers

Veterinary World editorial team sincerely like to thank all of our reviewers who contributed to peer review for the journal in 2025.

**List of countries (76 countries):** Algeria, Argentina, Armenia, Australia, Austria, Bangladesh, Belgium, Brazil, Bulgaria, Burkina Faso, Canada, Chile, China, Christmas Island, Colombia, Cote D’Ivoire, Croatia, Czech Republic, Denmark, Ecuador, Egypt, Ethiopia, France, Gabon, Germany, Greece, Hungary, India, Indonesia, Iran, Iraq, Ireland, Italy, Japan, Jordan, Kazakhstan, Kenya, Korea, Kuwait, Latvia, Lebanon, Libya, Lithuania, Malaysia, Mexico, Morocco, Myanmar, Netherlands, Nigeria, Oman, Pakistan, Peru, Philippines, Poland, Portugal, Romania, Russia, Saudi Arabia, South Africa, South Korea, Spain, Sweden, Switzerland, Taiwan, Tanzania, Thailand, Tunisia, Turkey, Uganda, Ukraine, United Kingdom, Uruguay, USA, Vietnam, Zambia, Zimbabwe.

1. A Aygun Turkey

2. A. Demeli Turkey

3. A. Kahyani Iran

4. A.K. Bulashev Kazakhstan

5. Aamir Ali Pakistan

6. Aan Awaludin Indonesia

7. Abhijit S. Deshmukh India

8. Abolfazl Ghaniei Iran

9. Agung Irawan USA

10. Ahmed Adel Elsayed Egypt

11. Ahmed Kamr USA

12. Ahmed Mohamed Mabrouk Saudi Arabia

13. Ahmed S Abdoon Egypt

14. Ahmed Saad Ahmed Hassaneen Egypt

15. Alberto Elmi Italy

16. Aldi Salman Indonesia

17. Alec Evans Kenya

18. Alejandro Hidalgo Chile

19. Alfredo Medrano Mexico

20. Ali Anil Suleymanoglu Turkey

21. Alireza Seidavi Iran

22. Alok Kumar India

23. Alvaro Diazbadillo USA

24. Amir Nahal Algeria

25. Amira Taman Egypt

26. Amitava Dey India

27. Amjad Islam Aqib Pakistan

28. Anas Abdullah Hamad Iraq

29. Anastasia V. Simakova Russia

30. Andrea Marchegiani Italy

31. Andrea Paolini Italy

32. Andreas Mershin USA

33. Andreia Coutinho Facin Brazil

34. Andrey Panitskiy Kazakhstan

35. Anihouvi G. H. Anihouvi Belgium

36. Anna Nowaczek Poland

37. Antonio J. Vallecillo Ecuador

38. Anupama Dr. Mukherjee India

39. Anut Chantiratikul Thailand

40. Archana Jain India

41. Arda Sozcu Turkey

42. Artem Blagodatski Russia

43. Asghar Abbas Pakistan

44. Ashish Chopra India

45. Ashok Kumar India

46. Ashwani Kumar India

47. Asim Faraz Pakistan

48. Aulfat Tahseen Yaseen Iraq

49. Ayman Abdel-Aziz Swelum Egypt

50. Ayrton Breno Pimenta Brazil

51. Aysu Deirmenci Dkaya Turkey

52. B. Asadi USA

53. Badjibassa Akounda Burkina Faso

54. Bahja Al Riyami Oman

55. Bamidele T. Odumosu Nigeria

56. Banan Khalid Al-Baggou Iraq

57. Baratang Alison Lubisi South Africa

58. Bartosz Kiero324;Czyk Poland

59. Batoul Mohamed Izzularab Egypt

60. Belal S Obeidat Jordan

61. Belgin SIRIKEN Turkey

62. Bernad, Elena Romania

63. Betina Boneva Marutsova Bulgaria

64. Bhanusupriya USA

65. Blazej Nowak Poland

66. Brandon Flatgard USA

67. Brbara Martnmaldonado Spain

68. Breno Fernando Martins De Almeida Brazil

69. Buket Er Demirhan Turkey

70. Bulent Ekiz Turkey

71. C. Usugamonroy Colombia

72. Carmen Solcan Romania

73. Cassia Regina Oliveira Santos Brazil

74. Cenk Er Turkey

75. Chaidate Inchaisri Thailand

76. Chandan Cswri Prakash India

77. Chao-Nan Lin Taiwan

78. Charleata A Carter USA

79. Cher Chien Lau Malaysia

80. Chitradevi Somasundaram India

81. Christin Kleinsorgen Germany

82. Clarissa Silveira Luiz Vaz Brazil

83. Cláudia Braga Pereira Bento Brazil

84. Claudia Giannetto Italy

85. Csaba Hancz Hungary

86. Daniel Ionut Berean Romania

87. Dariusz Kucharczyk Poland

88. Darwin Yovanny Hernndez Colombia

89. Darya Khalid Mahmood Iraq

90. Debasis Nayak India

91. Deepak Gola India

92. Deepali Gopal Kalambhe India

93. Deepan Kishore USA

94. Deepti Narang India

95. Denys Demianenko Ukraine

96. Derrick Noah Sentamu Kenya

97. Dhruv Nitinkumar Desai USA

98. Di Cataldo Sophia Argentina

99. Dilip Kumar Swain India

100. Dimar Sari Wahyuni Indonesia

101. Dirceu Guilherme De Souza Ramos Brazil

102. Dmitrii E. Aleshin Russia

103. Dmitry G. Deryabin Russia

104. Dominique Ouédraogo Burkina Faso

105. Dragica Salamon Croatia

106. Dwi Priyowidodo Indonesia

107. E Uzun Yaylac Turkey

108. E.E. Onbaşilar Turkey

109. Eaftekhar Ahmed Rana Bangladesh

110. Edson Ireeta Munanura Uganda

111. Eduardo Ghiggi Brazil

112. Eduardo Jorge Boeri Argentina

113. Elena Garde Chile

114. Elena Harran Lebanon

115. Elif Çelik Turkey

116. Elise L. Mckean USA

117. Elizabeth Salinasestrella Mexico

118. Eloiza May Galon Philippines

119. Eman Dhahir Arif Iraq

120. Eman S. Elwakil Egypt

121. Emily Haddy UK

122. Erma Safitri Indonesia

123. Essam Almadaly Egypt

124. Essam S. Soliman Egypt

125. Estelle Manguin France

126. Ethayathulla Abdul Samath India

127. Ewa Sellkubiak Poland

128. Fabio Cardoso Leal Freitas Portugal

129. Fabrizio Bertelloni Italy

130. Fatma M.A. Yousseff Egypt

131. Fei Zhao China

132. Fereshteh Mehri Iran

133. Fernando De Freitas Fernandes Brazil

134. Fernando Sánchez-Dávila Mexico

135. Firmain N. Yokoly Cote D’Ivoire

136. Florence D Tarfa, David Tarfa Nigeria

137. Fouad Kasim Mohammad Iraq

138. Francesco Serrapica Italy

139. Francisco Bez Jos Baez Uruguay

140. G. Radhika India

141. G. Taru Sharma India

142. Gabriel Vieira Ramos Brazil

143. Gamil Sayed Zeedan Egypt

144. Gautham Kolluri India

145. Genevieve Andre-Fontaine France

146. Ghadeer Sabah Bustani Iraq

147. Gianmarco Ferrara Italy

148. Gianmarco Ferrara Italy

149. Girma Zewdie Delelegn Ethiopia

150. H. Bahbahani Kuwait

151. Haini Pao Taiwan

152. Hani H. Albaadani Saudi Arabia

153. Hany Elsheikha UK

154. Hany Youssef Hassan Egypt

155. Harvie P. Portugaliza Philippines

156. Hasan Aydin Turkey

157. Hasan MEYDAN Turkey

158. Hashim Hadi Aljebory Iraq

159. Hasria Alang Indonesia

160. Haval Mohammed Khalid Iraq

161. Haytham Senbill Egypt

162. Hazem Shaheen Egypt

163. Hechuan Wang China

164. Hector Gabriel Varela Argentina

165. Heliot Zarza Mexico

166. Hodallah H. Ahmed Egypt

167. Hongbin Si Christmas Island

168. Hristina Zlatanovatenisheva Bulgaria

169. Huaji Qiu China

170. Huansheng Wu China

171. Hui-Chen Guo China

172. Huiling Sun China

173. Hung Luong USA

174. Hung Pham Vietnam

175. Iang Schroniltgen Rondon Barragan Colombia

176. Ilham Zaidi India

177. Ilona Kaszak Poland

178. Imen Ayedboussema Tunisia

179. Indah Amalia Amri Indonesia

180. Indrasen Chauhan India

181. Ionel Alexandru Checherita Romania

182. Ionel D. BONDOC Romania

183. Ismael Hernandez Avalos Mexico

184. İsmail Hakkı Tekiner Turkey

185. Ivo Sirakov Bulgaria

186. Jabulani Nkululeko Ngcobo South Africa

187. Jamal Gharekhani Iran

188. Jane M Morrell Sweden

189. Janet E. Alexander United Kingdom

190. Jareerat Aiemsaard Thailand

191. Jennifer Cole United Kingdom

192. Jianjun Wang China

193. Jianye Wang China

194. Jidang Chen China

195. Jihoi Moon Korea

196. Jinu Manoj India

197. Jiunling Wang Taiwan

198. Jnos Dgi Romania

199. Joab Malanda Osotsi Hungary

200. João Simões Portugal

201. Joaquin Gadea Spain

202. John F. Mee Ireland

203. Joice San Andres Philippines

204. Jonathan T Bvunzawabaya USA

205. Joong Sun Kim South Korea

206. Jorge A. Maldonado-Jáquez Mexico

207. Jose Francisco Francisco Rivera Benitez Mexico

208. Jose Luis Campos Spain

209. Joseph Cyrus Parambeth Canada

210. Joshua Mbanga Zimbabwe

211. Juan Carlos Sepulveda Arias Colombia

212. Juan Wang China

213. Jubeda Begum India

214. Julian Ruiz Saenz Colombia

215. Kannan Thandavan Arthanari India

216. Karim El Sabrout Egypt

217. Kasim Sakran Abass Iraq

218. Kavous Solhjoo Iran

219. Kekungu Puro India

220. Kezhou Cai China

221. Kimdiep Tran Vietnam

222. Koushlesh Ranjan India

223. Krpasha Dixon United Kingdom

224. Laurent F. Bonnac USA

225. Lawrence Mugisha Uganda

226. Layla Elmajdoub Libya

227. Lenin Lenin Aguirre Riofrio Ecuador

228. Leonardo Cabrera Mexico

229. Letizia Sinagra Italy

230. Liga Kovalcuka Latvia

231. Ligi Milesh USA

232. Liliana Aguilar-Marcelino Mexico

233. Linous Munsimbwe Zambia

234. Lonneke L. Ijsseldijk Netherlands

235. Luca Todini Italy

236. M Saminathan India

237. M Suman Kumar India

238. M Zamri Saad Malaysia

239. M. Teresa Teresa Tejedor Spain

240. Machrumnizar MACHRUMNIZAR Indonesia

241. Magdi M. Waheed Saudi Arabia

242. Mahdi Ali Abdullah Iraq

243. Mahipal Singh Sankhla India

244. Mahmoud Mohey Elhaig Egypt

245. Majid Fasihi Harandi Iran

246. Malgorzata Gajewska Poland

247. Manal Hadi Ghaffoori Kanaan Iraq

248. Manlin Wei China

249. Marcia Raquel Pegoraro De Macedo Italy

250. Márcio Moura-Alves Portugal

251. Marcus Antnio Rossi Feliciano Brazil

252. María Denisse Montoya-Flores Mexico

253. Maria Florencia Gallelli Argentina

254. Maria Maklakova Mexico

255. Maria Paola Cassarani Italy

256. Mariola Bochniarz Poland

257. Martina Felici Italy

258. Martina Gerken Germany

259. Marwan FADHIL Turkey

260. Maryam Dadar Iran

261. Matthew B. Wolf USA

262. Md Hafizur Rahman Bangladesh

263. Md Zakir Hassan Bangladesh

264. Md. Ahaduzzaman Bangladesh

265. Md. Tanvir Rahman Bangladesh

266. Megha Agarwal USA

267. Mengmeng Zhao China

268. Menno Holzhauer Netherlands

269. Miguel Tavares Tavares Pereira Switzerland

270. Mihaela Niculae Romania

271. Mikhail Syromyatnikov Russia

272. Mirosław Mariusz Michalski Poland

273. Mohamed Abomosallam Egypt

274. Mohamed Benaddou Morocco

275. Mohamed Donia USA

276. Mohamed E. Abd Elhack Egypt

277. Mohamed LOUNIS Algeria

278. Mohamed Salah Ayyat Egypt

279. Mohamed Wael Abd El-Azim Egypt

280. Mohammad Jokar Canada

281. Mohammed Baqur S Alshuhaib Iraq

282. Mohammed El-Houadfi Morocco

283. Mohammed Fouad El Basuini Egypt

284. Mohammed Gagaoua France

285. Mohammed S. Alhajj Saudi Arabia

286. Mohan Singh Thakur India

287. Mona Ahmed Elzamkan Egypt

288. Mona Said Mahmoud Egypt

289. Morakot Kaewthamasorn Thailand

290. Mostafa Helal Egypt

291. Mosua Tavassoli Iran

292. Moustafa Mohamed Zeitoun Egypt

293. Mr Marques Portugal

294. Muhammad Abdul Basit Pakistan

295. Muhammad Asif Rasheed Pakistan

296. Muhammad Mubashir China

297. Muhammad Thohawi Elziyad Purnama Indonesia

298. N. W. Khalil Egypt

299. Nadine Ndilimabaka Gabon

300. Nadlirotun Luthfi Indonesia

301. Nalinee Tuntivanich Thailand

302. Nanshan Qi China

303. Nantawan Therdthai Thailand

304. Nataliia Mokhnachova Ukraine

305. Natapol Pumipuntu Thailand

306. Nattawooti Sthitmatee Thailand

307. Nattha Jariyapamornkoon Thailand

308. Naveeda Akhtar Qureshi Pakistan

309. Naveen Kumar B.T. India

310. Nazmul Haque USA

311. Neil Vezeau USA

312. Nesreen Allam Tantawy Allam Egypt

313. Niluh Putu Dharmayanti Indonesia

314. Noha Mahmoud Fahmy Hassan Egypt

315. Noppason Pangprasit Thailand

316. Nozipho Lindiwe Khumalo South Africa

317. Nuchsupha Sunthamala Thailand

318. Nur Azira Tukiran Malaysia

319. Nurliyana Mohamad Malaysia

320. Obert Tada South Africa

321. Om Prakash Choudhary India

322. Ondĭej Krunt Czech Republic

323. Opeyemi Samson Osuntokun Nigeria

324. Osman Gaafar Mahgoub Canada

325. Pablo Jesús Marín-García Spain

326. Panagiotis E Simitzis Greece

327. Paulraj Philip Samuel India

328. Pavel Kic Czech Republic

329. Peerapol Sukon Thailand

330. Pernille Tvedennyborg Denmark

331. Peter Ayodeji Idowu South Africa

332. Petr Rauer Czech Republic

333. Petras Prakas Lithuania

334. Piya Temviriyanukul Thailand

335. Pınar Peker Akalın Turkey

336. Pınar Şanlıbaba; Turkey

337. Pornchai Pornpapom Thailand

338. Pourouchottamane R. India

339. Priyanka Uppe India

340. Prof Elsayed Eldeeb Egypt

341. Prof. Dr. Muhammad Ijaz Pakistan

342. Pururava Sharma India

343. Qizhong Zhang China

344. R. Rajasekhar India

345. R.Saravanan Ramasamy India

346. R.Umaya Suganthi India

347. Rafael Cardoso Carvalho Brazil

348. Rajat Jangir India

349. Rajeev Ranjan India

350. Rajendran K. V. India

351. Randall Lockwood USA

352. Rao Zahid Abbas Pakistan

353. Rasa Binkien Lithuania

354. Ratchaneegorn Mapanao Thailand

355. Ratika Rahmasari Indonesia

356. Ravi Kanth Reddy Poonooru India

357. Reenu Singh Tanwar USA

358. Remil L. Galay Philippines

359. Renata Assis Casagrande Brazil

360. Ricardo Tafuri Brando Brazil

361. Riyaz Anjum Sherasiya India

362. Robert J. Geraghty USA

363. Roberta Perego Italy

364. Rodrigo Alberto Jerez Ebensperger Spain

365. Rogério Ribeiro Vicentini Brazil

366. Rukhsana Amin Runa Bangladesh

367. Rumbidzai Mangoyi Zimbabwe

368. Runjun Yang China

369. Ruxandra Pavel Romania

370. Ryou Tanaka Japan

371. S.K. Mondal India

372. Saber Raeghi Iran

373. Sachin Kumar India

374. Safdar Ali Pakistan

375. Saganuwan Alhaji Saganuwan Nigeria

376. Sajid Ali Pakistan

377. Sakshaleni Rajendiran Malaysia

378. Salvador Eduardo Acevedomonroy Mexico

379. Samir Dey India

380. Sana A M Elgayar Egypt

381. Sankar Venkatachalam India

382. Sanyuan Huang Taiwan

383. Saran Promsai Thailand

384. Saravanan Subramaniam India

385. Satenik Kharatyan Armenia

386. Saw Bawm Myanmar

387. Sayerh Fatimazahra Morocco

388. Seid Kassaw Ali Ethiopia

389. Sena Ardicli Switzerland

390. Serkan Sayiner Turkey

391. Seyedeh Mahdieh Khoshnazar Iran

392. Shahid Nazir Australia

393. Shaimaa A. Mohamed Egypt

394. Shaimaa H. Negm Egypt

395. Shameeran Salman Ismael Iraq

396. Shekhar Ramchandra Bhagwat India

397. Shengfan Wang Taiwan

398. Shujaat Hussain Pakistan

399. Shumaila Taskeen India

400. Shyma K P India

401. Siju Susan Jacob India

402. Simon Peter Musinguzi Uganda

403. Sindhu Abraham India

404. Sintija Jonova Latvia

405. Siti Zubaidah Ramanoon Malaysia

406. Sleyman Ilek Turkey

407. Socheat Sieng Australia

408. Sorawat Thongsahuan Thailand

409. Soul Washaya Zimbabwe

410. Suchawan Pornsukarom Thailand

411. Sucheeva Junnu Thailand

412. Sudhakar Pralhad Awandkar India

413. Sumera Sajjad Pakistan

414. Suporn Thongyuan Thailand

415. Suresh H Basagoudanavar India

416. Takalani Judas Mpofu South Africa

417. Tanko Polycarp Nwunuji Nigeria

418. Thankgod E. Onyiche Nigeria

419. Thavasimuthu Citarasu India

420. The Nguyen USA

421. Theofanis Aravanis Greece

422. Thotsapol Thomrongsuwannakij Thailand

423. Todd Hsu Taiwan

424. Tong Bu China

425. Toshiro Arai Japan

426. Trker Sava Turkey

427. Usama T. Mahmoud Egypt

428. Usman Ibe Michael Uganda

429. V.V.S.Suryanarayana India

430. Valeria Silva Alvarez Uruguay

431. Valery Silvery Sonola Tanzania

432. Vanessa S. Cruz Brazil

433. Vasfiye KADER ESEN Turkey

434. Vicente Rodríguezestvez Spain

435. Victor Alexander Temoche Peru

436. Victor Alves Carneiro Brazil

437. Vijay Pal Singh India

438. Vijaya Kumar Anumolu India

439. Vimbai Gobvu Zimbabwe

440. Vincenzo Tufarrelli Italy

441. Vishal S Suthar India

442. Vukka Chengalva Rayulu India

443. Wael A. Khalil Egypt

444. Walter Conca Austria

445. Weerapol Taweenan Thailand

446. Widya Paramita Lokapirnasari Indonesia

447. Wisam S Hassan Iraq

448. Wisnu Nurcahyo Indonesia

449. Xiaole Qi China

450. Yangchun Cao China

451. Yetti Marlida Indonesia

452. Yudi Adinata Indonesia

453. Yun Ma China

454. Z. Husin Malaysia

455. Zahid Naseer Pakistan

456. Zaven Karalyan Armenia

457. Zeki Erol Turkey

458. Zhigang Wang China

459. Zhiyong Fan China

460. Ziam Hocine Algeria

